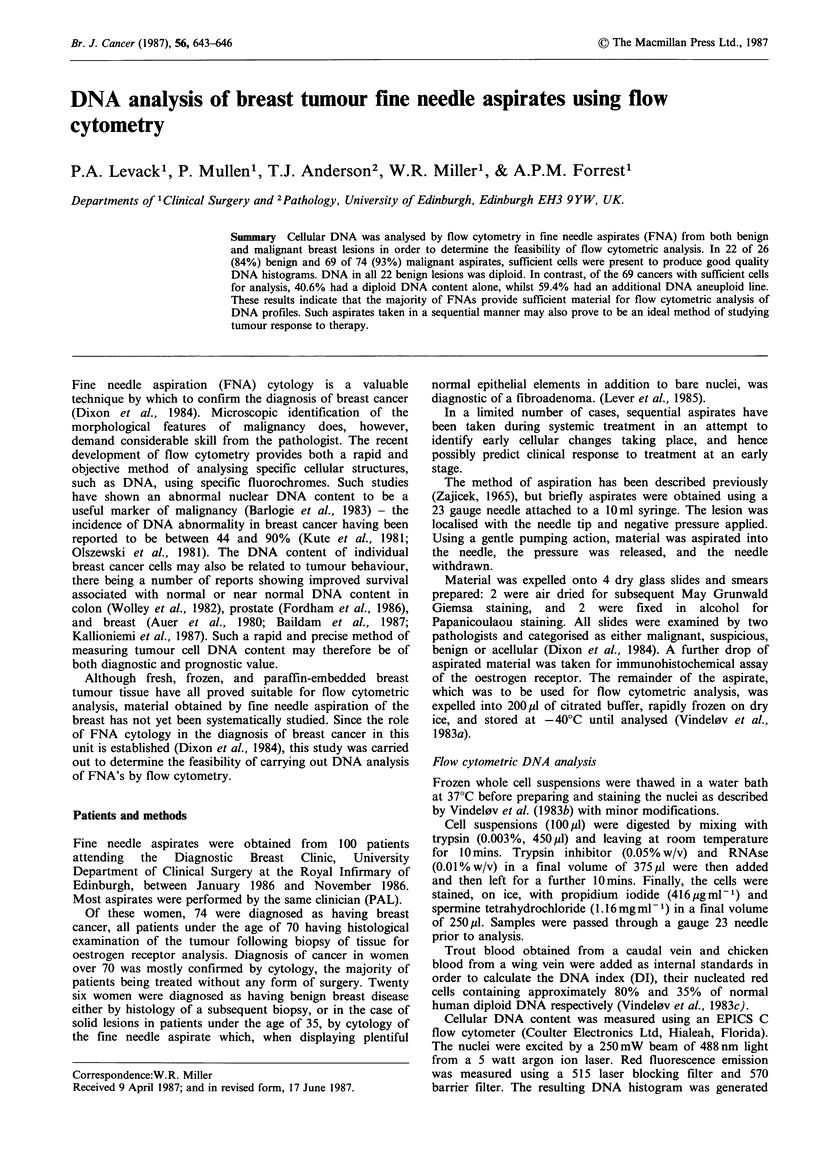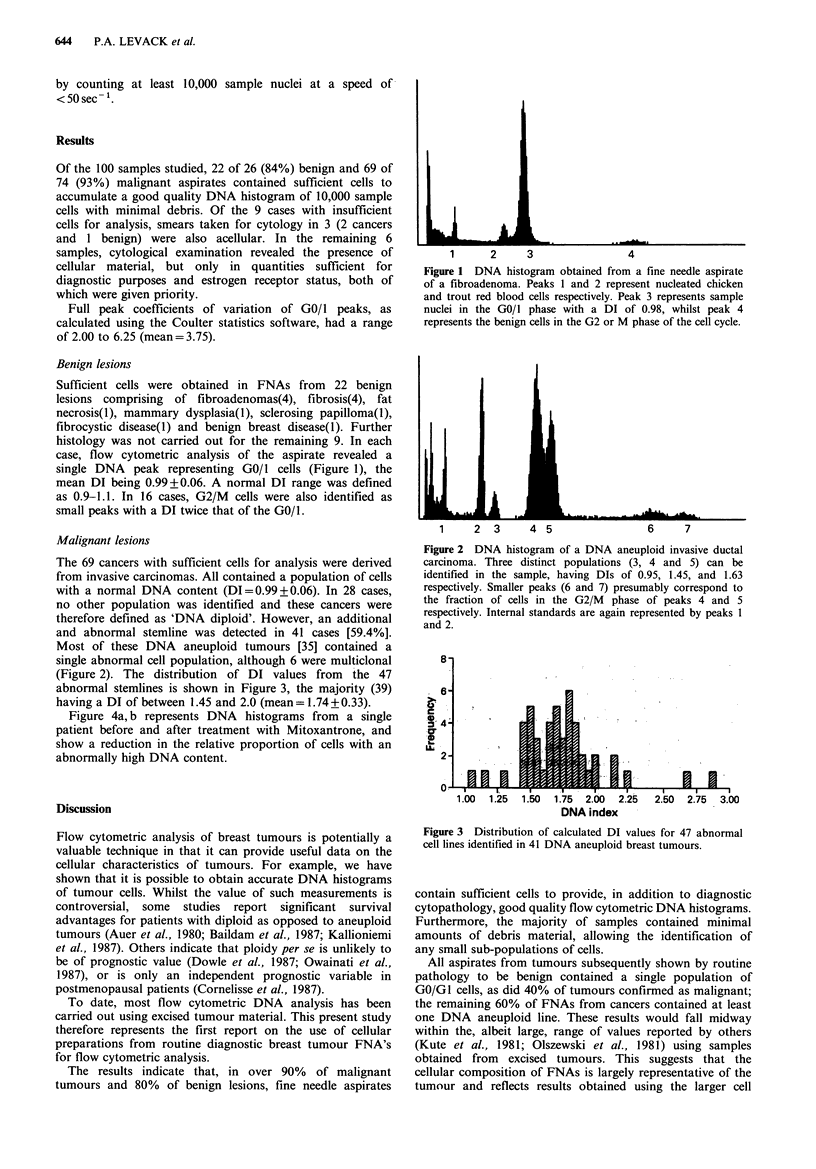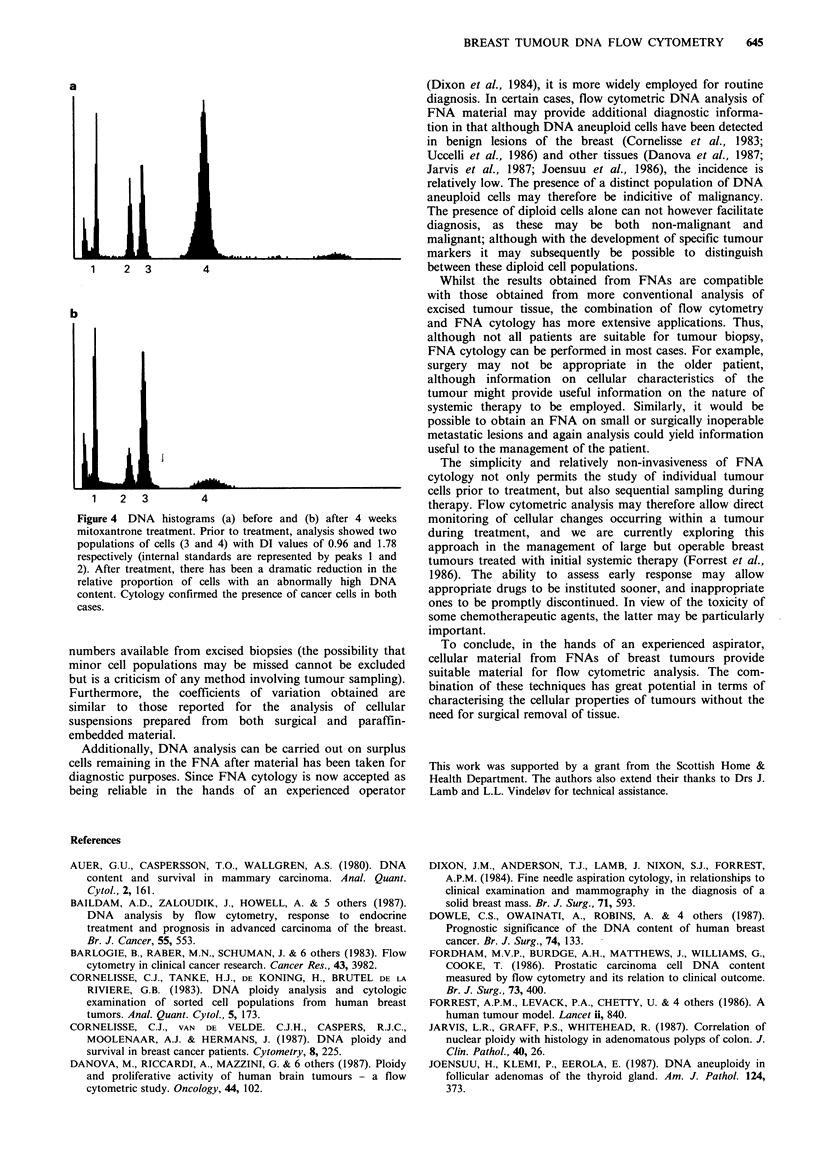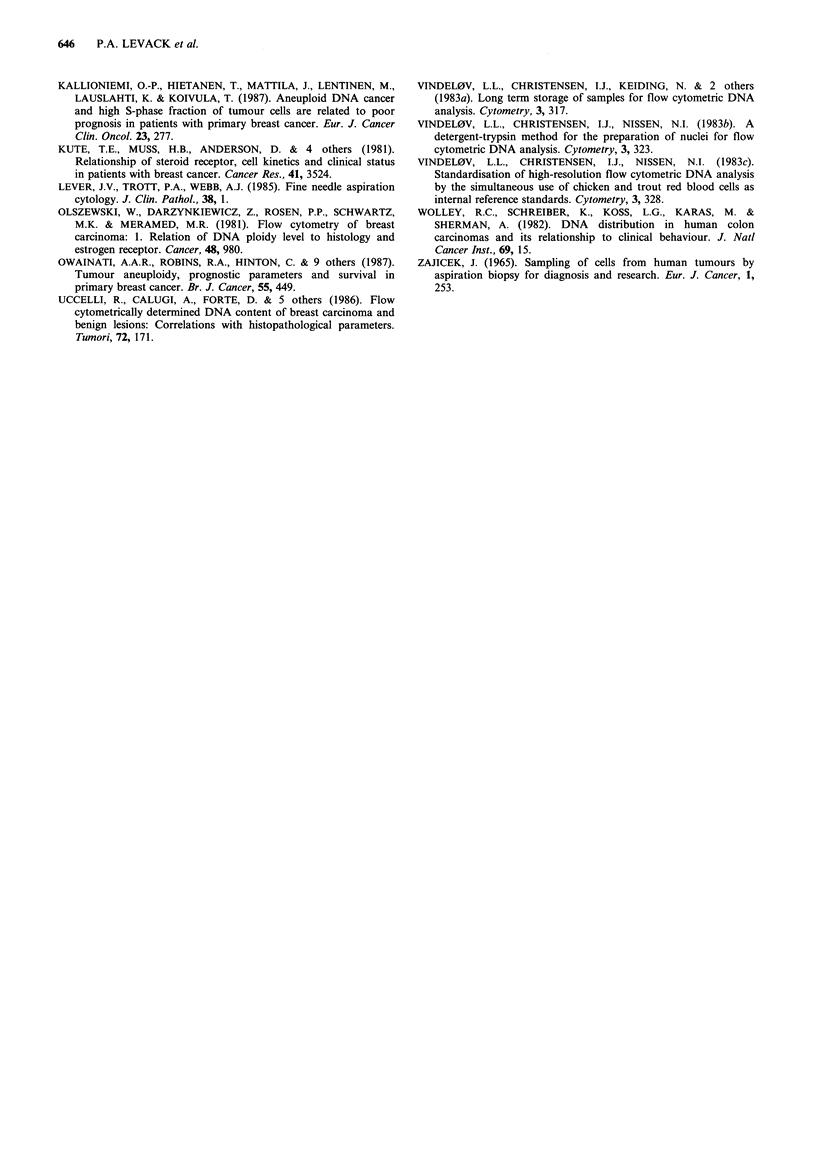# DNA analysis of breast tumour fine needle aspirates using flow cytometry.

**DOI:** 10.1038/bjc.1987.259

**Published:** 1987-11

**Authors:** P. A. Levack, P. Mullen, T. J. Anderson, W. R. Miller, A. P. Forrest

**Affiliations:** Department of Clinical Surgery, University of Edinburgh, UK.

## Abstract

Cellular DNA was analysed by flow cytometry in fine needles aspirates (FNA) from both benign and malignant breast lesions in order to determine the feasibility of flow cytometric analysis. In 22 of 26 (84%) benign and 69 of 74 (93%) malignant aspirates, sufficient cells were present to produce good quality DNA histograms. DNA in all 22 benign lesions was diploid. In contrast, of the 69 cancers with sufficient cells for analysis, 40.6% had a diploid DNA content alone, whilst 59.4% had an additional DNA aneuploid line. These results indicate that the majority of FNAs provide sufficient material for flow cytometric analysis of DNA profiles. Such aspirates taken in a sequential manner may also prove to be an ideal method of studying tumour response to therapy.


Br. J. Cancer (1987), 56, 643–646 © The Macmillan Press Ltd., 1987

### DNA analysis of breast tumour fine needle aspirates using flow cytometry

P.A. Levack[1], P. Mullen[1], T.J. Anderson[2], W.R. Miller[1], & A.P.M. Forrest[1]

*Departments of [1]Clinical Surgery and [2]Pathology, University of Edinburgh, Edinburgh EH3 9YW, UK.*

**Summary** Cellular DNA was analysed by flow cytometry in fine needle aspirates (FNA) from both benign and malignant breast lesions in order to determine the feasibility of flow cytometric analysis. In 22 of 26 (84%) benign and 69 of 74 (93%) malignant aspirates, sufficient cells were present to produce good quality DNA histograms. DNA in all 22 benign lesions was diploid. In contrast, of the 69 cancers with sufficient cells for analysis, 40.6% had a diploid DNA content alone, whilst 59.4% had an additional DNA aneuploid line. These results indicate that the majority of FNAs provide sufficient material for flow cytometric analysis of DNA profiles. Such aspirates taken in a sequential manner may also prove to be an ideal method of studying tumour response to therapy.

Fine needle aspiration (FNA) cytology is a valuable technique by which to confirm the diagnosis of breast cancer (Dixon *et al.*, 1984). Microscopic identification of the morphological features of malignancy does, however, demand considerable skill from the pathologist. The recent development of flow cytometry provides both a rapid and objective method of analysing specific cellular structures, such as DNA, using specific fluorochromes. Such studies have shown an abnormal nuclear DNA content to be a useful marker of malignancy (Barlogie *et al.*, 1983) – the incidence of DNA abnormality in breast cancer having been reported to be between 44 and 90% (Kute *et al.*, 1981; Olszewski *et al.*, 1981). The DNA content of individual breast cancer cells may also be related to tumour behaviour, there being a number of reports showing improved survival associated with normal or near normal DNA content in colon (Wolley *et al.*, 1982), prostate (Fordham *et al.*, 1986), and breast (Auer *et al.*, 1980; Baildam *et al.*, 1987; Kallioniemi *et al.*, 1987). Such a rapid and precise method of measuring tumour cell DNA content may therefore be of both diagnostic and prognostic value.

Although fresh, frozen, and paraffin-embedded breast tumour tissue have all proved suitable for flow cytometric analysis, material obtained by fine needle aspiration of the breast has not yet been systematically studied. Since the role of FNA cytology in the diagnosis of breast cancer in this unit is established (Dixon *et al.*, 1984), this study was carried out to determine the feasibility of carrying out DNA analysis of FNA's by flow cytometry.

### Patients and methods

Fine needle aspirates were obtained from 100 patients attending the Diagnostic Breast Clinic, University Department of Clinical Surgery at the Royal Infirmary of Edinburgh, between January 1986 and November 1986. Most aspirates were performed by the same clinician (PAL).

Of these women, 74 were diagnosed as having breast cancer, all patients under the age of 70 having histological examination of the tumour following biopsy of tissue for oestrogen receptor analysis. Diagnosis of cancer in women over 70 was mostly confirmed by cytology, the majority of patients being treated without any form of surgery. Twenty six women were diagnosed as having benign breast disease either by histology of a subsequent biopsy, or in the case of solid lesions in patients under the age of 35, by cytology of the fine needle aspirate which, when displaying plentiful normal epithelial elements in addition to bare nuclei, was diagnostic of a fibroadenoma. (Lever *et al.*, 1985).

In a limited number of cases, sequential aspirates have been taken during systemic treatment in an attempt to identify early cellular changes taking place, and hence possibly predict clinical response to treatment at an early stage.

The method of aspiration has been described previously (Zajicek, 1965), but briefly aspirates were obtained using a 23 gauge needle attached to a 10 ml syringe. The lesion was localised with the needle tip and negative pressure applied. Using a gentle pumping action, material was aspirated into the needle, the pressure was released, and the needle withdrawn.

Material was expelled onto 4 dry glass slides and smears prepared: 2 were air dried for subsequent May Grunwald Giemsa staining, and 2 were fixed in alcohol for Papanicoulaou staining. All slides were examined by two pathologists and categorised as either malignant, suspicious, benign or acellular (Dixon *et al.*, 1984). A further drop of aspirated material was taken for immunohistochemical assay of the oestrogen receptor. The remainder of the aspirate, which was to be used for flow cytometric analysis, was expelled into 200 $\mu$l of citrated buffer, rapidly frozen on dry ice, and stored at −40°C until analysed (Vindeløv *et al.*, 1983*a*).

*Flow cytometric DNA analysis*

Frozen whole cell suspensions were thawed in a water bath at 37°C before preparing and staining the nuclei as described by Vindeløv *et al.* (1983*b*) with minor modifications.

Cell suspensions (100 $\mu$l) were digested by mixing with trypsin (0.003%, 450 $\mu$l) and leaving at room temperature for 10 mins. Trypsin inhibitor (0.05% w/v) and RNAse (0.01% w/v) in a final volume of 375 $\mu$l were then added and then left for a further 10 mins. Finally, the cells were stained, on ice, with propidium iodide (416 $\mu g\,ml^{-1}$) and spermine tetrahydrochloride (1.16 $mg\,ml^{-1}$) in a final volume of 250 $\mu$l. Samples were passed through a gauge 23 needle prior to analysis.

Trout blood obtained from a caudal vein and chicken blood from a wing vein were added as internal standards in order to calculate the DNA index (DI), their nucleated red cells containing approximately 80% and 35% of normal human diploid DNA respectively (Vindeløv *et al.*, 1983*c*).

Cellular DNA content was measured using an EPICS C flow cytometer (Coulter Electronics Ltd, Hialeah, Florida). The nuclei were excited by a 250 mW beam of 488 nm light from a 5 watt argon ion laser. Red fluorescence emission was measured using a 515 laser blocking filter and 570 barrier filter. The resulting DNA histogram was generated

Correspondence: W.R. Miller
Received 9 April 1987; and in revised form, 17 June 1987.

by counting at least 10,000 sample nuclei at a speed of $< 50\,sec^{-1}$.

### Results

Of the 100 samples studied, 22 of 26 (84%) benign and 69 of 74 (93%) malignant aspirates contained sufficient cells to accumulate a good quality DNA histogram of 10,000 sample cells with minimal debris. Of the 9 cases with insufficient cells for analysis, smears taken for cytology in 3 (2 cancers and 1 benign) were also acellular. In the remaining 6 samples, cytological examination revealed the presence of cellular material, but only in quantities sufficient for diagnostic purposes and estrogen receptor status, both of which were given priority.

Full peak coefficients of variation of G0/1 peaks, as calculated using the Coulter statistics software, had a range of 2.00 to 6.25 (mean = 3.75).

### *Benign lesions*

Sufficient cells were obtained in FNAs from 22 benign lesions comprising of fibroadenomas(4), fibrosis(4), fat necrosis(1), mammary dysplasia(1), sclerosing papilloma(1), fibrocystic disease(1) and benign breast disease(1). Further histology was not carried out for the remaining 9. In each case, flow cytometric analysis of the aspirate revealed a single DNA peak representing G0/1 cells (Figure 1), the mean DI being $0.99 \pm 0.06$. A normal DI range was defined as 0.9–1.1. In 16 cases, G2/M cells were also identified as small peaks with a DI twice that of the G0/1.

### *Malignant lesions*

The 69 cancers with sufficient cells for analysis were derived from invasive carcinomas. All contained a population of cells with a normal DNA content ($DI = 0.99 \pm 0.06$). In 28 cases, no other population was identified and these cancers were therefore defined as 'DNA diploid'. However, an additional and abnormal stemline was detected in 41 cases [59.4%]. Most of these DNA aneuploid tumours [35] contained a single abnormal cell population, although 6 were multiclonal (Figure 2). The distribution of DI values from the 47 abnormal stemlines is shown in Figure 3, the majority (39) having a DI of between 1.45 and 2.0 ($mean = 1.74 \pm 0.33$).

Figure 4a, b represents DNA histograms from a single patient before and after treatment with Mitoxantrone, and show a reduction in the relative proportion of cells with an abnormally high DNA content.

**Figure 1** DNA histogram obtained from a fine needle aspirate of a fibroadenoma. Peaks 1 and 2 represent nucleated chicken and trout red blood cells respectively. Peak 3 represents sample nuclei in the G0/1 phase with a DI of 0.98, whilst peak 4 represents the benign cells in the G2 or M phase of the cell cycle.

**Figure 2** DNA histogram of a DNA aneuploid invasive ductal carcinoma. Three distinct populations (3, 4 and 5) can be identified in the sample, having DIs of 0.95, 1.45, and 1.63 respectively. Smaller peaks (6 and 7) presumably correspond to the fraction of cells in the G2/M phase of peaks 4 and 5 respectively. Internal standards are again represented by peaks 1 and 2.

**Figure 3** Distribution of calculated DI values for 47 abnormal cell lines identified in 41 DNA aneuploid breast tumours.

### Discussion

Flow cytometric analysis of breast tumours is potentially a valuable technique in that it can provide useful data on the cellular characteristics of tumours. For example, we have shown that it is possible to obtain accurate DNA histograms of tumour cells. Whilst the value of such measurements is controversial, some studies report significant survival advantages for patients with diploid as opposed to aneuploid tumours (Auer *et al.*, 1980; Baildam *et al.*, 1987; Kallioniemi *et al.*, 1987). Others indicate that ploidy *per se* is unlikely to be of prognostic value (Dowle *et al.*, 1987; Owainati *et al.*, 1987), or is only an independent prognostic variable in postmenopausal patients (Cornelisse *et al.*, 1987).

To date, most flow cytometric DNA analysis has been carried out using excised tumour material. This present study therefore represents the first report on the use of cellular preparations from routine diagnostic breast tumour FNA's for flow cytometric analysis.

The results indicate that, in over 90% of malignant tumours and 80% of benign lesions, fine needle aspirates contain sufficient cells to provide, in addition to diagnostic cytopathology, good quality flow cytometric DNA histograms. Furthermore, the majority of samples contained minimal amounts of debris material, allowing the identification of any small sub-populations of cells.

All aspirates from tumours subsequently shown by routine pathology to be benign contained a single population of G0/G1 cells, as did 40% of tumours confirmed as malignant; the remaining 60% of FNAs from cancers contained at least one DNA aneuploid line. These results would fall midway within the, albeit large, range of values reported by others (Kute *et al.*, 1981; Olszewski *et al.*, 1981) using samples obtained from excised tumours. This suggests that the cellular composition of FNAs is largely representative of the tumour and reflects results obtained using the larger cell

**Figure 4** DNA histograms (a) before and (b) after 4 weeks mitoxantrone treatment. Prior to treatment, analysis showed two populations of cells (3 and 4) with DI values of 0.96 and 1.78 respectively (internal standards are represented by peaks 1 and 2). After treatment, there has been a dramatic reduction in the relative proportion of cells with an abnormally high DNA content. Cytology confirmed the presence of cancer cells in both cases.

numbers available from excised biopsies (the possibility that minor cell populations may be missed cannot be excluded but is a criticism of any method involving tumour sampling). Furthermore, the coefficients of variation obtained are similar to those reported for the analysis of cellular suspensions prepared from both surgical and paraffin-embedded material.

Additionally, DNA analysis can be carried out on surplus cells remaining in the FNA after material has been taken for diagnostic purposes. Since FNA cytology is now accepted as being reliable in the hands of an experienced operator (Dixon *et al.*, 1984), it is more widely employed for routine diagnosis. In certain cases, flow cytometric DNA analysis of FNA material may provide additional diagnostic information in that although DNA aneuploid cells have been detected in benign lesions of the breast (Cornelisse *et al.*, 1983; Uccelli *et al.*, 1986) and other tissues (Danova *et al.*, 1987; Jarvis *et al.*, 1987; Joensuu *et al.*, 1986), the incidence is relatively low. The presence of a distinct population of DNA aneuploid cells may therefore be indicitive of malignancy. The presence of diploid cells alone can not however facilitate diagnosis, as these may be both non-malignant and malignant; although with the development of specific tumour markers it may subsequently be possible to distinguish between these diploid cell populations.

Whilst the results obtained from FNAs are compatible with those obtained from more conventional analysis of excised tumour tissue, the combination of flow cytometry and FNA cytology has more extensive applications. Thus, although not all patients are suitable for tumour biopsy, FNA cytology can be performed in most cases. For example, surgery may not be appropriate in the older patient, although information on cellular characteristics of the tumour might provide useful information on the nature of systemic therapy to be employed. Similarly, it would be possible to obtain an FNA on small or surgically inoperable metastatic lesions and again analysis could yield information useful to the management of the patient.

The simplicity and relatively non-invasiveness of FNA cytology not only permits the study of individual tumour cells prior to treatment, but also sequential sampling during therapy. Flow cytometric analysis may therefore allow direct monitoring of cellular changes occurring within a tumour during treatment, and we are currently exploring this approach in the management of large but operable breast tumours treated with initial systemic therapy (Forrest *et al.*, 1986). The ability to assess early response may allow appropriate drugs to be instituted sooner, and inappropriate ones to be promptly discontinued. In view of the toxicity of some chemotherapeutic agents, the latter may be particularly important.

To conclude, in the hands of an experienced aspirator, cellular material from FNAs of breast tumours provide suitable material for flow cytometric analysis. The combination of these techniques has great potential in terms of characterising the cellular properties of tumours without the need for surgical removal of tissue.

This work was supported by a grant from the Scottish Home & Health Department. The authors also extend their thanks to Drs J. Lamb and L.L. Vindeløv for technical assistance.

### References

AUER, G.U., CASPERSSON, T.O., WALLGREN, A.S. (1980). DNA content and survival in mammary carcinoma. *Anal. Quant. Cytol.*, **2**, 161.

BAILDAM, A.D., ZALOUDIK, J., HOWELL, A. & 5 others (1987). DNA analysis by flow cytometry, response to endocrine treatment and prognosis in advanced carcinoma of the breast. *Br. J. Cancer*, **55**, 553.

BARLOGIE, B., RABER, M.N., SCHUMAN, J. & 6 others (1983). Flow cytometry in clinical cancer research. *Cancer Res.*, **43**, 3982.

CORNELISSE, C.J., TANKE, H.J., DE KONING, H., BRUTEL DE LA RIVIERE, G.B. (1983). DNA ploidy analysis and cytologic examination of sorted cell populations from human breast tumors. *Anal. Quant. Cytol.*, **5**, 173.

CORNELISSE, C.J., VAN DE VELDE. C.J.H., CASPERS, R.J.C., MOOLENAAR, A.J. & HERMANS, J. (1987). DNA ploidy and survival in breast cancer patients. *Cytometry*, **8**, 225.

DANOVA, M., RICCARDI, A., MAZZINI, G. & 6 others (1987). Ploidy and proliferative activity of human brain tumours – a flow cytometric study. *Oncology*, **44**, 102.

DIXON, J.M., ANDERSON, T.J., LAMB, J. NIXON, S.J., FORREST, A.P.M. (1984). Fine needle aspiration cytology, in relationships to clinical examination and mammography in the diagnosis of a solid breast mass. *Br. J. Surg.*, **71**, 593.

DOWLE, C.S., OWAINATI, A., ROBINS, A. & 4 others (1987). Prognostic significance of the DNA content of human breast cancer. *Br. J. Surg.*, **74**, 133.

FORDHAM, M.V.P., BURDGE, A.H., MATTHEWS, J., WILLIAMS, G., COOKE, T. (1986). Prostatic carcinoma cell DNA content measured by flow cytometry and its relation to clinical outcome. *Br. J. Surg.*, **73**, 400.

FORREST, A.P.M., LEVACK, P.A., CHETTY, U. & 4 others (1986). A human tumour model. *Lancet* **ii**, 840.

JARVIS, L.R., GRAFF, P.S., WHITEHEAD, R. (1987). Correlation of nuclear ploidy with histology in adenomatous polyps of colon. *J. Clin. Pathol.*, **40**, 26.

JOENSUU, H., KLEMI, P., EEROLA, E. (1987). DNA aneuploidy in follicular adenomas of the thyroid gland. *Am. J. Pathol.* **124**, 373.

KALLIONIEMI, O.-P., HIETANEN, T., MATTILA, J., LENTINEN, M., LAUSLAHTI, K. & KOIVULA, T. (1987). Aneuploid DNA cancer and high S-phase fraction of tumour cells are related to poor prognosis in patients with primary breast cancer. *Eur. J. Cancer Clin. Oncol.* **23**, 277.

KUTE, T.E., MUSS, H.B., ANDERSON, D. & 4 others (1981). Relationship of steroid receptor, cell kinetics and clinical status in patients with breast cancer. *Cancer Res.*, **41**, 3524.

LEVER, J.V., TROTT, P.A., WEBB, A.J. (1985). Fine needle aspiration cytology. *J. Clin. Pathol.*, **38**, 1.

OLSZEWSKI, W., DARZYNKIEWICZ, Z., ROSEN, P.P., SCHWARTZ, M.K. & MERAMED, M.R. (1981). Flow cytometry of breast carcinoma: 1. Relation of DNA ploidy level to histology and estrogen receptor. *Cancer*, **48**, 980.

OWAINATI, A.A.R., ROBINS, R.A., HINTON, C. & 9 others (1987). Tumour aneuploidy, prognostic parameters and survival in primary breast cancer. *Br. J. Cancer*, **55**, 449.

UCCELLI, R., CALUGI, A., FORTE, D. & 5 others (1986). Flow cytometrically determined DNA content of breast carcinoma and benign lesions: Correlations with histopathological parameters. *Tumori*, **72**, 171.

VINDELØV, L.L., CHRISTENSEN, I.J., KEIDING, N. & 2 others (1983*a*). Long term storage of samples for flow cytometric DNA analysis. *Cytometry*, **3**, 317.

VINDELØV, L.L., CHRISTENSEN, I.J., NISSEN, N.I. (1983*b*). A detergent-trypsin method for the preparation of nuclei for flow cytometric DNA analysis. *Cytometry*, **3**, 323.

VINDELØV, L.L., CHRISTENSEN, I.J., NISSEN, N.I. (1983*c*). Standardisation of high-resolution flow cytometric DNA analysis by the simultaneous use of chicken and trout red blood cells as internal reference standards. *Cytometry*, **3**, 328.

WOLLEY, R.C., SCHREIBER, K., KOSS, L.G., KARAS, M. & SHERMAN, A. (1982). DNA distribution in human colon carcinomas and its relationship to clinical behaviour. *J. Natl Cancer Inst.*, **69**, 15.

ZAJICEK, J. (1965). Sampling of cells from human tumours by aspiration biopsy for diagnosis and research. *Eur. J. Cancer*, **1**, 253.